# Supplementary material for: Exon Elongation Added Intrinsically Disordered Regions to the Encoded Proteins and Facilitated the Emergence of the Last Eukaryotic Common Ancestor
Source: Mol Biol Evol. 2022 Dec 19;40(1):msac272. doi: 10.1093/molbev/msac272 (PMC9825244; doi:10.1093/molbev/msac272)
Supplement: msac272_Supplementary_Data [file msac272_supplementary_data.pdf]

Supplementary Material for
Exon Elongation Added Intrinsically Disordered Regions to the Encoded Proteins and Facilitated the Emergence of the Last Eukaryotic Common Ancestor

Satoshi Fukuchi[1], Tamotsu Noguchi[2], Hiroto Anbo[1], and Keiichi Homma[1, *]

[1] Program for Information Systems, Division of Informatics, Bioengineering and Bioscience, Maebashi Institute of Technology; 460-1 Kamisadori-machi, Maebashi-shi, 371-0816 Japan

[2] Pharmaceutical Education Research Center, Meiji Pharmaceutical University; 2-522-1 Noshio, Kiyose, Tokyo, 204-8588 Japan

[*]**Corresponding author:** khomma@maebashi-it.ac.jp

4 supplementary figures and 7 supplementary tables

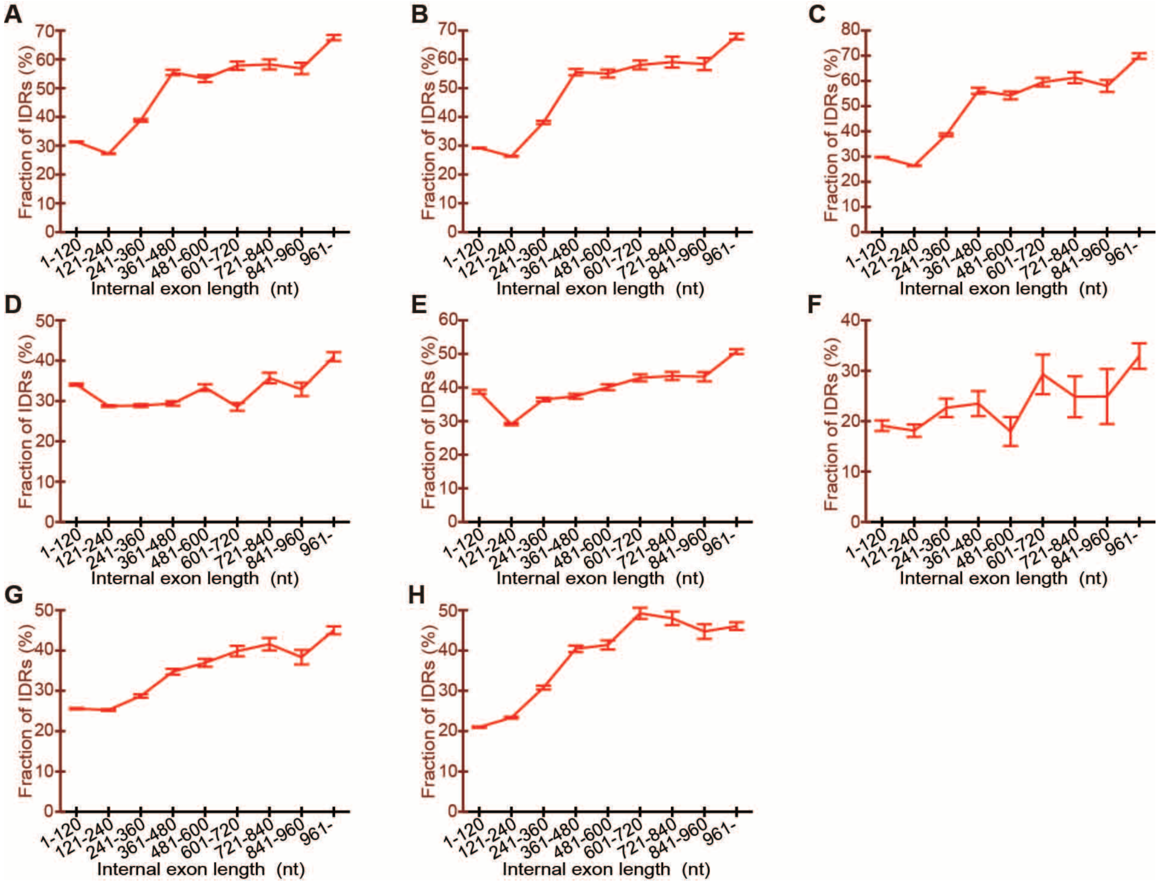

Supplementary Fig. S1. DICHOT results show that long internal exons tend to encode IDRs. The mean fractions of IDRs encoded by the internal exons in the length rages with SEMs are displayed by line graphs. The IDRs were predicted by DICHOT and are presented as in Fig. 1.

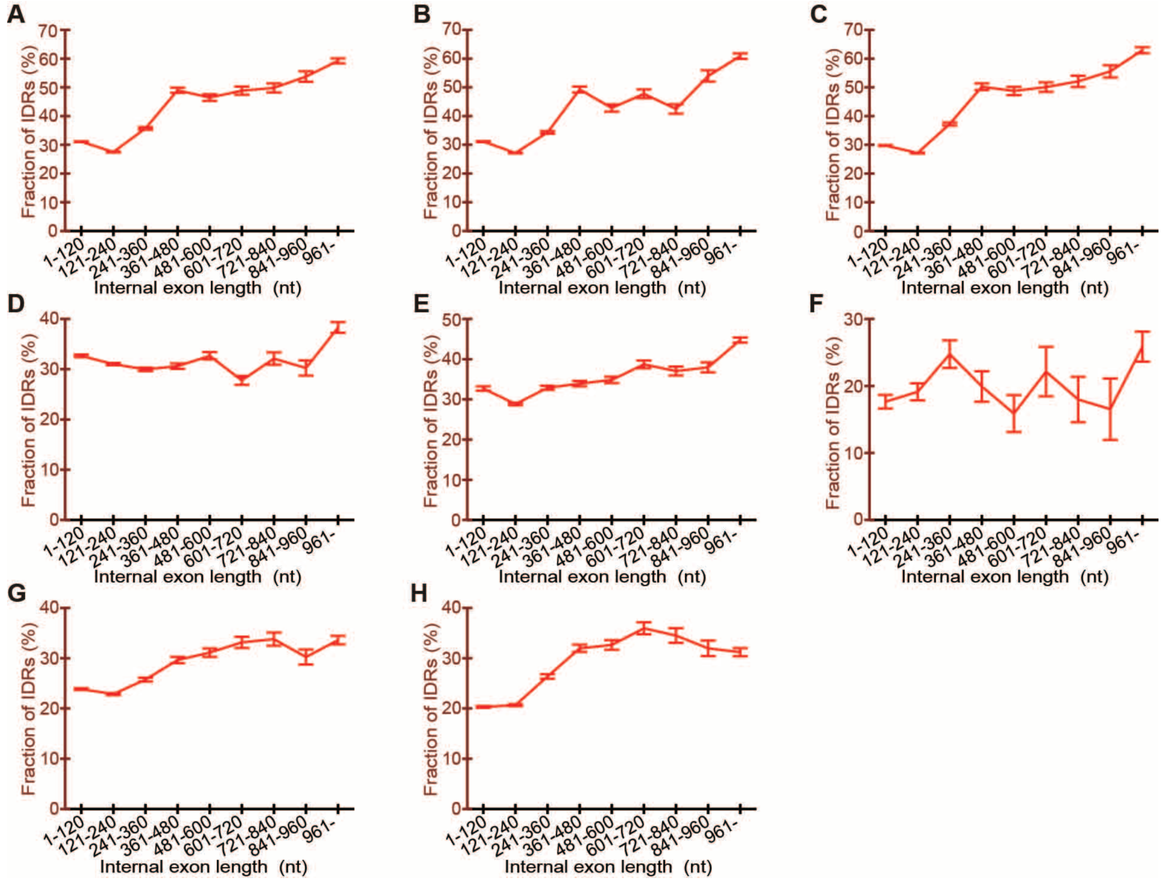

Supplementary Fig. S2. POODLE-L results show that long internal exons tend to encode IDRs. The mean fractions of IDRs encoded by the internal exons in the length rages with SEMs are displayed by line graphs. The IDRs were predicted by POODLE-L and are shown as in Fig. 1.

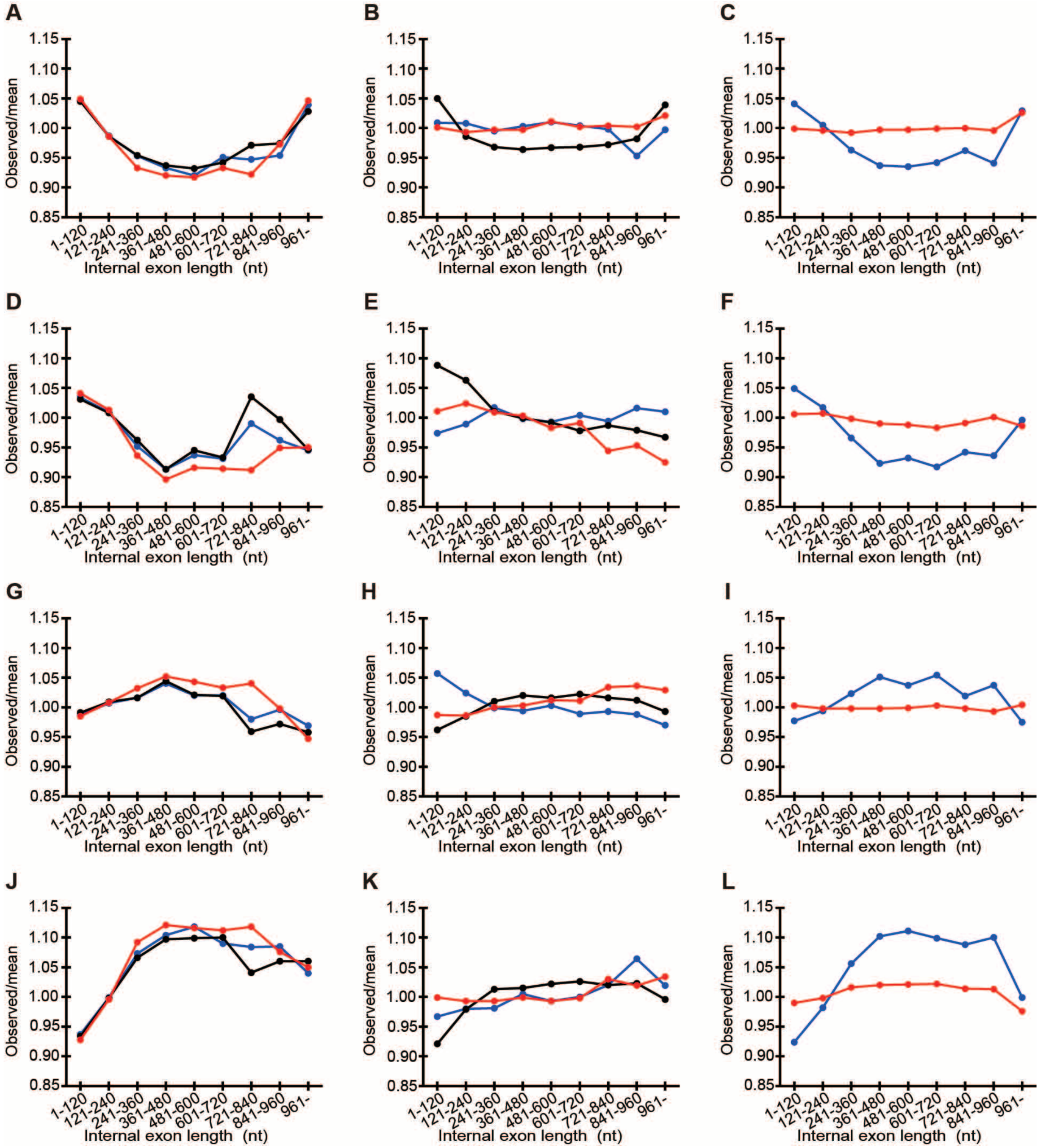

Supplementary Fig. S3. Long internal exons in most but not all species have high cytidine contents. The observed frequency of a nucleotide in the internal exons in each length bin is divided by the average frequency of the nucleotide in all the internal exons in the species. The ratios in each species coloured are shown as line graphs. The nucleotides plotted are *(A-C)* adenine, (*D-F*) thymidine, (*G-I*) guanosine, and (*J-L*) cytidine. (*A*, *D*, *G*, and *J*) *H. sapiens* (red), *M. musculus* (black), and *R. norvegicus* (blue), (*B*, *E*, *H*, and *K*) *C. elegans* (red), *D. melanogaster* (black), and *S. pombe* (blue), (*C*, *F*, *I*, and *L*) *A. thaliana* (red) and *O. sativa* (blue).

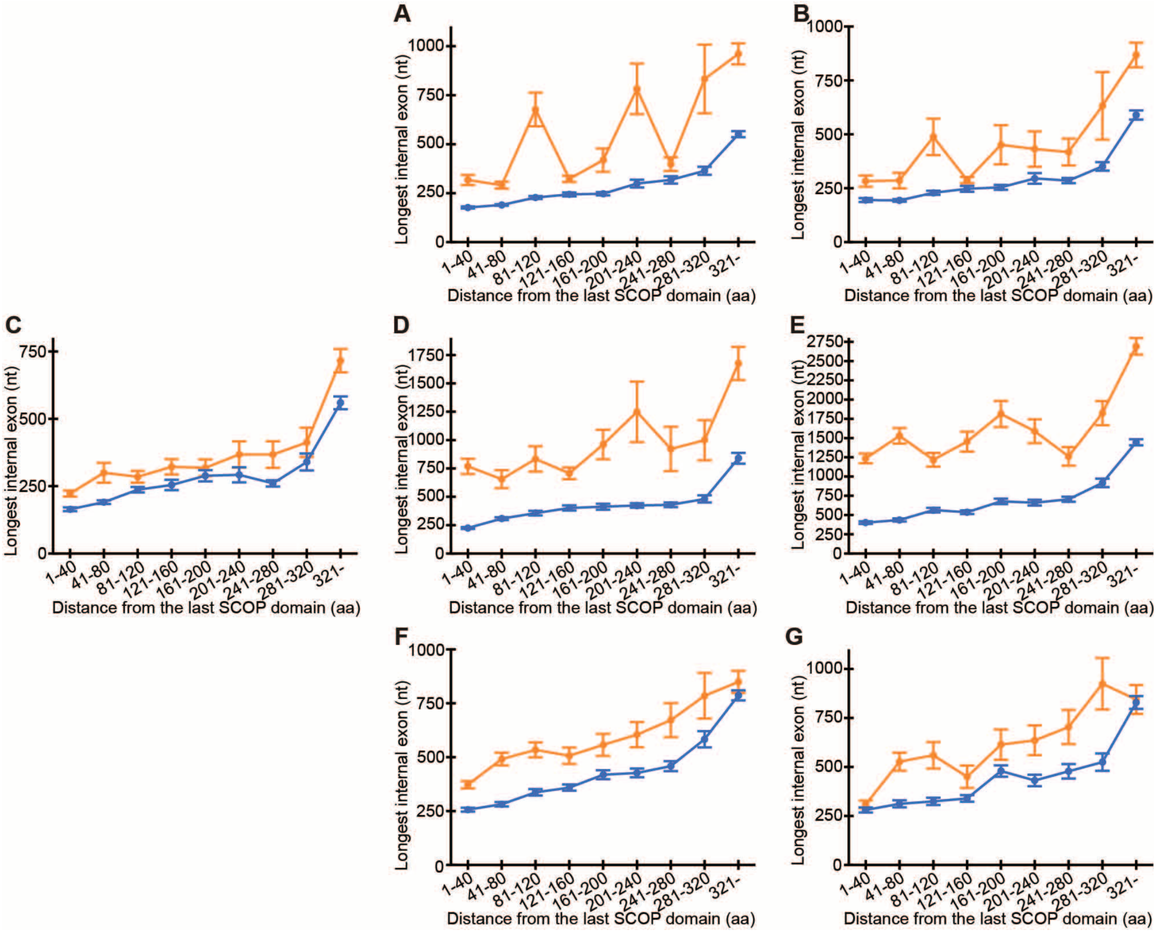

Supplementary Fig. S4. A SCOP domain followed by a long internal exon frequently has another SCOP domain downstream even if only half of SCOP domains are randomly selected and used for analysis. The mean lengths and SEMs of the longest internal exons in group A (orange) and B (blue) segments are shown as solid lines if the numbers of both groups are in excess of 20. The species plotted are (*A*) *H. sapiens*, (*B*) *M. musculus*, (*C*) *R. norvegicus*, (*D*) *C. elegans*, (*E*) *D. melanogaster*, (*F*) *A. thaliana*, and (*G*) *O. sativa*.

Supplementary Table S1. The number of samples and correlation coefficients (*R*s) between internal exon length and per cent IDRs.

Species	Hsa	Mmu	Rno	Cel	Dme	Spo	Ath	Osa
Sample number	187,484	178,114	166,917	87,985	37,952	2,785	100,776	85,468
R by DISOPRED3	0.098	0.109	0.113	0.065	0.130	0.146	0.099	0.152
R by DICHOT	0.074	0.087	0.086	0.007*	0.093	0.073	0.071	0.100
R by POODLE-L	0.066	0.065	0.077	0.009*	0.097	0.020*	0.048	0.074

*Not significantly different from 0 at $P<0.001$, while the rest are significantly different from 0 at $P<0.001$.

Supplementary Table S2. The number of genes with maximum internal exon lengths in each range.

Maximum internal exon length (nt)	Hsa	Mmu	Rno	Cel	Dme	Spo	Ath	Osa
1-120	3,655	1,474	858	616	712	365	2,982	2,448
121-240	11,665	9,581	7,163	3,287	2,140	345	7,184	7,141
241-360	3,948	3,364	2,427	3,174	1,735	169	4,112	3,734
361-480	1,461	1,104	826	2,030	1,508	130	2,058	1,796
481-600	941	724	520	1,207	1,279	66	1,323	1,039
601-720	674	578	417	889	975	58	800	716
721-840	501	423	287	499	773	53	550	506
841-960	369	323	228	314	656	36	430	439
961-	1,600	1,354	885	731	2,894	145	1,374	1,541

Supplementary Table S3. The expected fractions (%) of constrained exons and Spearman's correlation coefficients (r_s's).

Internal exon length (nt)	HSA	MMU	RNO	CEL	EME	SPO	ATH	OSA
1-120	33.96	34.20	34.29	23.00	28.59	25.42	36.29	36.19
121-240	29.39	29.63	29.69	21.25	24.53	21.92	32.44	31.81
241-360	21.63	21.61	22.07	19.15	20.21	20.21	26.87	24.05
361-480	19.36	17.97	17.79	16.99	18.86	18.74	23.89	19.81
481-600	15.60	15.75	15.58	14.46	16.74	20.88	19.03	15.87
601-720	17.54	17.09	17.30	13.87	16.28	16.06	17.87	16.72
721-840	18.82	19.72	19.38	15.30	17.63	22.65	17.35	13.50
841-960	19.76	18.18	19.72	15.07	17.65	30.99	20.17	14.58
961-	16.83	16.09	15.69	14.71	16.77	15.41	21.29	16.51
r_s between internal exon length and observed fraction	-0.93	-0.93	-0.95	-0.95	-0.98	-1.00	-1.00	-0.97
r_s between internal exon length and observed/expected ratio	-0.92	-0.92	-0.92	-0.93	-0.97	-0.95	-1.00	-0.97

Supplementary Table S4. The number of inserted segments.

Internal exon length (nt)	Mmu	Rno
1-120	6753	4426
121-240	1863	930
241-360	1809	470
361-480	974	209
481-600	632	177
601-720	225	103
721-840	86	43
841-960	924	50
961-	7446	537

Supplementary Table S5. The number of segments in each range of distance from the last SCOP domain.

Group A	Hsa	Mmu	Rno	Cel	Dme	Spo	Ath	Osa
1-40 aa	9925	6013	3873	1671	3433	74	2622	1868
41-80 aa	2751	1766	1108	601	1206	33	1045	743
81-120 aa	1738	1068	671	376	702	17	585	303
121-160 aa	1135	765	487	214	431	6	357	200
161-200 aa	763	488	320	143	279	2	190	143
201-240 aa	549	369	229	86	252	1	198	79
241-280 aa	430	248	172	107	191	5	144	67
281-320 aa	333	198	130	82	162	1	109	74
321 aa-	1679	1104	689	290	1048	10	262	162

Group B	Hsa	Mmu	Rno	Cel	Dme	Spo	Ath	Osa
1-40 aa	4025	2068	1315	1054	1094	65	2456	2024
41-80 aa	2120	1204	803	595	608	26	1044	794
81-120 aa	1462	830	564	410	417	16	798	595
121-160 aa	988	560	408	318	410	15	653	434
161-200 aa	732	450	279	237	344	10	472	275
201-240 aa	563	370	229	210	277	9	349	238
241-280 aa	472	315	217	152	259	8	285	170
281-320 aa	369	237	154	120	184	8	207	143
321 aa-	2029	1346	870	559	1146	23	799	455

Supplementary Table S6. The number of segments in each range of distance from the last SCOP domain if half of SCOP domains are randomly selected and used for analysis.

Group A	Hsa	Mmu	Rno	Cel	Dme	Spo	Ath	Osa
1-40 aa	2634	1556	1053	450	863	18	664	471
41-80 aa	860	506	349	171	373	6	259	201
81-120 aa	817	458	274	136	289	9	198	88
121-160 aa	540	364	200	92	224	5	128	77
161-200 aa	419	270	151	81	176	2	89	50
201-240 aa	395	215	129	54	141	1	94	45
241-280 aa	270	152	100	53	117	2	62	42
281-320 aa	235	157	90	50	154	1	54	37
321 aa-	1266	836	495	238	770	9	169	100

Group B	Hsa	Mmu	Rno	Cel	Dme	Spo	Ath	Osa
1-40 aa	2166	1110	707	515	601	39	1305	1117
41-80 aa	1275	774	500	323	414	17	637	499
81-120 aa	1069	645	372	276	342	14	580	412
121-160 aa	800	417	348	229	282	15	490	330
161-200 aa	635	403	256	173	294	8	342	229
201-240 aa	534	332	246	199	239	6	278	207
241-280 aa	474	327	215	147	196	5	258	152
281-320 aa	418	228	161	92	180	7	225	138
321 aa-	2522	1655	1086	563	1255	24	919	518

Supplementary Table S7. List of programs used.

Species	Hsa	Mmu	Rno	Cel
Exons	hallexon3.c	mallexon3.c	nallexon3.c	callexon3.c
Non-redundant exons	halluniqexon3.c	malluniqexon3.c	nalluniqexon3.c	calluniqexon3.c
Non-redundant internal exons	halluniqexon3A.c	malluniqexon3A.c	nalluniqexon3A.c	calluniqexon3A.c
Internal exons with coding ranges	hallexon3M.c	mallexon3M.c	nallexon3M.c	callexon3M.c
Non-redundant internal exons with coding ranges	halluniqexon3M.c	malluniqexon3M.c	nalluniqexon3M.c	calluniqexon3M.c
Nonredundant exons with classification	halluniqexon4AB.c	malluniqexon4AB.c	nalluniqexon4AB.c	calluniqexon4AB.c
ORF list of cDNAs		mcdnaorflist1.c	ncdnaorflist1.c	ccdnaorflist1.c
Abbreviated nonredundant exons with classification	habbcdnalist1.c	mabbcdnalist1.c	nabbcdnalist1.c	cabbcdnalist1.c
Protein ID-transcript ID correspondence		mptcorr1.c	tptcorr1.c	
%IDR by DISOPRED3 (fig. 1)	hgintexst6M.c	mgintexst6M.c	ngintexst6M.c	cgintexst6M.c
Internal exon length distribution (fig. 1)	hlongexgenes1.c	mlongexgenes1.c	nlongexgenes1.c	clongexgenes1.c
SCOP analyses (fig. 2)	hscopconstex3.c	mscopconstex3.c	nscopconstex3.c	cscopconstex3.c
MAFFT alignments		hmrmafft1.c	hmrmafft1.c	
Species specific regions		minsseg1.c	tinsseg1.c	

Insertion in internal exons		mrinsexon1G.c	trinsexon1G.c	
%IDR in insertion by DISOPRED3 (fig.3)		mrinsidr6G.c	trinsidr6G.c	
%IDR in insertion by POODLE-L (fig. 3)		mrinsidr4G.c	trinsidr4G.c	
%IDR in insertion by DICHOT (fig. 3)		mrinsidr5G.c	trinsidr5G.c	
%Constrained exons		minsconstex3.c	tiinsconstex3.c	
Longest internal exon downstream of the last SCOP domain (fig. 5, table S5)	hscopadd2AM.c	mscopadd2AM.c	nscopadd2AM.c	cscopadd2AM.c
GO frequency	hlongingo3.c	mlongingo3.c		
GO in genes with long internal exons	commonlongingo3.c	commonlongingo3.c		
GO in genes with extremely long internal eons	commonlongingo4.c	commonlongingo4.c		
fig. S1 data	hintexst5M.c	mintexst5M.c	nintexst5M.c	cintexst5M.c
fig. S2 data	hgintexst4M.c	mgintexst4MC.c	ngintexst4M.c	cgintexst4M.c
fig. S3 data	hexdnacomp1.c	mexdnacomp1.c	nexdnacomp1.c	cexdnacomp1.c
fig. S4 data	hscopadd3AM.c	mscopadd3AM.c	nscopadd3AM.c	cscopadd3AM.c
table S1 data	halluniqexon3A.c	malluniqexon3A.c	nalluniqexon3A.c	calluniqexon3A.c
table S2 data	hscopnumb2.c	mscopnumb2.c	nscopnumb2.c	cscopnumb2.c
table S3 data	hscopconstex3.c	mscopconstex3.c	nscopconstex3.c	cscopconstex3.c
table S4 data		minsconstex3.c	tinsconstex3.c	

Species	Dme	Spo	Ath	Osa
Exons	dallexon3.c	fallexon3.c	aallexon3.c	rallexon3.c
Non-redundant exons	dalluniqexon3.c	falluniqexon3.c	aalluniqexon3.c	ralluniqexon3.c
Non-redundant internal exons	dalluniqexon3A.c	falluniqexon3A.c	aalluniqexon3A.c	ralluniqexon3A.c
Internal exons with coding ranges	dallexon3M.c	fallexon3M.c	aallexon3M.c	rallexon3M.c
Non-redundant internal exons with coding ranges	dalluniqexon3M.c	falluniqexon3M.c	aalluniqexon3M.c	ralluniqexon3M.c
Nonredundant exons with classification	dalluniqexon4AB.c	falluniqexon4AB.c	aalluniqexon4AB.c	ralluniqexon4AB.c
ORF list of cDNAs	dcdnaorflist1.c	fcdnaorflist1.c	acdnaorflist1.c	rcdnaorflist1.c
Abbreviated nonredundant exons with classification	dabbcdnalist1.c	fabbcdnalist1.c	aabbcdnalist1.c	rabbcdnalist1.c
%IDR by DISOPRED3 (fig. 1)	dgintexst6M.c	fgintexst6M.c	agintexst6M.c	rgintexst6M.c
Internal exon length distribution (fig. 1)	dlongexgenes1.c	flongexgenes1.c	alongexgenes1.c	rlongexgenes1.c
SCOP analyses (fig. 2)	dscopconstex3.c	fscopconstex3.c	ascopconstex3.c	rscopconstex3.c
Longest internal exon downstream of the last SCOP domain (fig. 5, table S5)	dscopadd2AM.c	fscopadd2AM.c	ascopadd2AM.c	rscopadd2AM.c
GO frequency		flongingo3.c	alongingo3.c	
GO in genes with long		commonlongingo3.c	commonlongingo3.c	

internal exons				
GO in genes with extremely long internal eons		commonlongingo4.c	commonlongingo4.c	
fig. S1 data	dintexst5M.c	fintexst5M.c	aintexst5M.c	rintexst5M.c
fig. S2 data	dgintexst4M.c	fgintexst4M.c	agintexst4M.c	rgintexst4M.c
fig. S3 data	dexdnacomp1.c	fexdnacomp1.c	aexdnacomp1.c	rexdnacomp1.c
fig. S4 data	dscopadd3AM.c	fscopadd3AM.c	ascopadd3AM.c	rscopadd3AM.c
table S1 data	dalluniqexon3A.c	falluniqexon3A.c	aalluniqexon3A.c	ralluniqexon3A.c
table S2 data	dscopnumb2.c	fscopnumb2.c	ascopnumb2.c	rscopnumb2.c
table S3 data	dscopconstex3.c	fscopconstex3.c	ascopconstex3.c	rscopconstex3.c
